# Social Cognition through the Lens of Cognitive and Clinical Neuroscience

**DOI:** 10.1155/2018/4283427

**Published:** 2018-09-13

**Authors:** Maria Arioli, Chiara Crespi, Nicola Canessa

**Affiliations:** ^1^NEtS Center, Scuola Universitaria Superiore IUSS, Pavia, 27100, Italy; ^2^Cognitive Neuroscience Laboratory, ICS Maugeri, Pavia, 27100, Italy

## Abstract

Social cognition refers to a set of processes, ranging from perception to decision-making, underlying the ability to decode others' intentions and behaviors to plan actions fitting with social and moral, besides individual and economic considerations. Its centrality in everyday life reflects the neural complexity of social processing and the ubiquity of social cognitive deficits in different pathological conditions. Social cognitive processes can be clustered in three domains associated with (a) perceptual processing of social information such as faces and emotional expressions (social perception), (b) grasping others' cognitive or affective states (social understanding), and (c) planning behaviors taking into consideration others', in addition to one's own, goals (social decision-making). We review these domains from the lens of cognitive neuroscience, i.e., in terms of the brain areas mediating the role of such processes in the ability to make sense of others' behavior and plan socially appropriate actions. The increasing evidence on the “social brain” obtained from healthy young individuals nowadays constitutes the baseline for detecting changes in social cognitive skills associated with physiological aging or pathological conditions. In the latter case, impairments in one or more of the abovementioned domains represent a prominent concern, or even a core facet, of neurological (e.g., acquired brain injury or neurodegenerative diseases), psychiatric (e.g., schizophrenia), and developmental (e.g., autism) disorders. To pave the way for the other papers of this issue, addressing the social cognitive deficits associated with severe acquired brain injury, we will briefly discuss the available evidence on the status of social cognition in normal aging and its breakdown in neurodegenerative disorders. Although the assessment and treatment of such impairments is a relatively novel sector in neurorehabilitation, the evidence summarized here strongly suggests that the development of remediation procedures for social cognitive skills will represent a future field of translational research in clinical neuroscience.

## 1. Making Sense of Others' Behavior with Social Cognition

Social cognition refers to a set of neurocognitive processes underlying the individuals' ability to “*make sense of others' behavior*” as a crucial prerequisite of social interaction [1]. Such a complex ability entails a variety of skills, ranging from decoding social information (e.g., faces and emotional expressions) and drawing inferences on others' mental or affective states to making decisions consistent with social norms and others' welfare.

Social abilities emerge as early as 14 months [2], also in nonhuman species [3], and remain crucial for the lifespan [4]. Their centrality in everyday life is clearly shown by those conditions in which a social cognitive impairment results in a variety of adverse outcomes, e.g., mental [5] and physical [6] deficits, functional disability [7], unemployment [5], and more generally poor quality of life [8]. The last edition of the American Psychiatric Association's Diagnostic and Statistical Manual for Mental Disorders (DSM-5) has indeed introduced social cognition as one of the six main factors of neurocognitive functioning, impaired in different pathological conditions.

Social cognitive impairments are a prominent concern, or even a core facet, of several neurodegenerative (e.g., behavioral variant of frontotemporal dementia), neuropsychiatric (e.g., schizophrenia, major depressive disorder, and bipolar disorder), and neurodevelopmental (e.g., autism spectrum disorder and attention deficit hyperactivity disorder) conditions, and often occur after acute brain damage (e.g., traumatic brain injury and stroke) [9]. Moreover, such deficits are critical predictors of functional outcomes because they affect the ability to create and maintain interpersonal relationships, thereby removing their benefits in everyday life [7]. In this respect, the rewarding and healthy value of social interaction [10] is shown by growing evidence on the negative consequences of isolation in terms of morbidity and mortality [11–13]. Interestingly,* perceived* social isolation (i.e., loneliness) is a major risk factor for several diseases, including dementia, independent of objective social isolation [14].

In order to pave the way for other articles of this special issue on the social cognitive deficits associated with acquired brain injury, this review aims at providing an overview of the social brain and its main functions. We will pursue this goal by summarizing the main findings obtained within the research field popularly known as “social cognitive neuroscience” [15]. For explanatory purposes, the complexity of social cognition will be addressed in terms of its three main domains, i.e., social perception, social understanding, and decision-making in the social context. Each of these subjects, representing distinct—although strictly intertwined—sectors of social neuroscience, will be first addressed in terms of cognitive processes and their modulating variables and then with regard to the available* f*MRI evidence on their neural correlates. Since the consequences of brain damage on social cognitive performance might be confounded by aging effects, in the last section we will briefly summarize the main findings of a fast-growing literature concerned with age-related changes in different facets of social cognition. To complement the evidence on the effects of acquired brain injury presented in other articles of this issue, this section will also review few selected findings from a lively interdisciplinary research sector exploring social cognitive deficits in neurodegenerative disorders. To introduce the potential translational implications of research in social cognitive neuroscience, we conclude by discussing selected examples of social cognitive treatment protocols assessed in previous studies and the available meta-analytic evidence about their effectiveness.

## 2. Three Main Domains of Social Cognition

The ability to establish appropriate social interactions entails several distinct processes. First, the social agent must recognize the others as “living persons,” via the analysis of complex perceptual information including facial expressions, gestures, postures and body language, and voice, [16]. Once integrated, this information will represent the input for higher-level processes underlying a direct resonance to others' affective states (i.e., “empathy”) and/or the interpretation of their observable behaviors in terms of mental states and dispositions (i.e., “mentalizing” or “theory of mind” [17]). By modulating decision-making, the outcome of these processes will likely lead the observer to adapt her/his own social behavior [18]. This framework highlights the three key domains of social cognition which will be discussed in the next sections, i.e., social perception, social understanding, and social decision-making.

### 2.1. 

#### 2.1.1. Social Perception

A basic prerequisite of social cognition is the ability to distinguish between objects (whose behavior is completely explained by physical forces) and persons (characterized by inner experiences, such as motivations, reasons, and intentions, which make their behavior not completely predictable) (Fiske and Taylor, 2013) [19].

A related question in social cognitive neuroscience is whether social stimuli represent a qualitatively different perceptual category or rather the specificity of their neural processing can be reduced to “low-level” perceptual dimensions such as vividness, salience or familiarity (Fiske & Taylor, 2013). The former hypothesis fits with the centrality of social stimuli in human life, with their different functions being expressed at various levels of complexity: survival for the single individual, communication in dyads, social coordination in groups, and, finally, culture in institutions [20]. The prototypical example, in this respect, is represented by the neural processing of human faces [21], providing multifaceted information on both others' changeable characteristics such as emotions and intentions, and invariant features such as identity. The unique salience of human faces [22] is indeed considered to reflect their predictive power with respect to others' intentions and thus their potential consequence in social terms [23]. In line with this view, different experimental paradigms suggest that faces and objects undergo different styles of cognitive processing, i.e., holistic vs. part-based coding, respectively, with parts being integrated into a whole in upright but not inverted faces [24]. This evidence for the unique status of faces fits with the existence of a dedicated neural circuitry for this category of social stimuli, additionally showing stronger responses to upright than inverted faces [25].

In particular, the eyes represent the most dynamic and informative social stimulus, capturing our attention more than head/body movements and postures [26]. Gaze direction reveals overt attention shifts, and the informative value of another's eye-movement patterns with respect to her/his mental states explains why gaze perception is considered a crucial prerequisite of mentalizing [27]. Alongside gaze, also the emotional expressions produced by the contractions of facial muscles provide crucial social information [28, 29]. In addition to the obvious communicative valence of emotions (“*A radar and rapid response system, constructing and carrying meaning across the flow of experience*” [30]), it is important to stress their adaptive value for appraising experience and preparing to act in response to external stimuli. The popular Ekman and Friesen's (2003) facial action coding scheme (FACS) describes facial expressions as combinations of the action units characterizing different emotions. This model is based on the notion of a set of six basic universal emotions (happiness, anger, sadness, fear, disgust, and surprise) which all humans can express and recognize regardless of sociocultural effects [31]. It is worth mentioning that more recently, a similar proposal has been made for specific social emotions such as shame and embarrassment (Cordaro et al., 2017). On the other hand, available evidence on the role played by cultural rules on the processing of facial expression and interpretation of emotions strengthens an “interactionist perspective” taking into consideration both biological and social/cultural factors [32].

While facial expressions represent the most effective means for emotional communication, the latter can involve also the body [33] and the voice [34]. In the first case, bodily changes are related to the role of emotions in preparing to act in response to external stimuli. If different emotions involve specific patterns of body movement and posture, this information could support emotional decoding based on visuomotor analyses of body language. Evidence based on point-light displays indeed shows high accuracy in relating such a minimal information to the emotion expressed by a moving body [35]. In addition, voices reveal our feelings as well, through nonverbal vocalizations (e.g., laugh) and prosody. However, available evidence suggests that the voice conveys mostly unspecific facets of affective states, such as physiological arousal [36], but no clear cue to specific emotions. On the other hand, the combination of different features could contribute to distinguish emotions in spoken sentences [37], and there is evidence for intersubject reliability in emotional judgments based on vocalizations [38]. Moreover, although most studies have addressed the information provided by face, body, and voice in isolation, the typical co-occurrence of multiple input channels improves emotional decoding (Martinez et al., 2015).

According to the “Feedback hypothesis”, faces, voices, and bodies not only express but also influence emotional experiences, because the production of facial expressions, sounds, and postures results in related sensory feedback which in turn modulates the intensity of feelings [39]. The latter would be thus enhanced by the expression of a congruent emotion and decreased either by the inhibition of a congruent emotion or by the expression of an incongruent emotion [40]. This hypothesis suggests a tight relationship between the perceptual and “private” facets of emotional processing, which fits with recent evidence on emotion perception. Several theoretical speculations and empirical investigations on this subject revolve around the notion of “embodied simulation.” That is, a mirror-like mechanism [41] is considered to provide a direct link between the first- and third-person experiences and thus access to the meaning of others' actions and emotions [42]. In this perspective, mirroring the others' facial emotional expressions, via the engagement of the corresponding motor circuits and muscular contractions (i.e., mimicry; [43, 44]), underpins a direct and experiential grasp of their meaning [45].

The notion of embodiment, however, has been also proposed to underlie even cognitive phenomena exceeding perception and action. According to the “embodied cognition” framework [46], all cognitive representations and operations would be fundamentally grounded in their physical sensory-motor context [47]. Even our semantic knowledge would be ultimately represented, at the neural level, in the sensory-motor systems underlying our direct experience with the world (Niedenthal, 2007), so that semantic representations of objects or events involve (some of) the brain sensory-motor states associated with their direct experience (Barsalou, 2008). This approach strongly departs from associative network models, considering memory as a web of semantic concepts that describe objects and events [48] in terms of basic units represented by propositions [49]. In the latter framework, any object would be represented in memory by a set of descriptive propositions, interconnected by associative links made through experience. The engagement of an emotion unit would spread activity in this interconnected web [50], thus increasing the accessibility to words and memories associated with the target emotion [51]. In the embodied cognition framework, instead, even the somehow “abstract” facets of emotional processing, such as those representing the affective value of an object brought to memory, involves reactivating the motor programs and feelings associated with its direct sensorimotor experience [52]. The latter would then provide an experiential access to the meaning of concepts, including their affective features.

#### 2.1.2. Neural Correlates of Social Perception

The fast growth of social cognitive neuroscience is providing increasing evidence on the brain networks subserving the different domains previously described, and the available data nowadays allow to fractionate the social brain in distinct sets of areas associated with relatively specific functions. We will focus on the neural processing of visual stimuli, representing the richest source of information in everyday social life as well as in the available literature.

The first nodes of the neural pathways underlying the processing of visual social stimuli involve the occipitotemporal cortex, where distinct brain regions have been associated with a preliminary decomposition of the visual scene into different categories and particularly faces (Occipital Face Area (OFA) in the inferior occipital gyrus and Fusiform Face Area (FFA) in the fusiform gyrus; [53]) and bodies or body-parts (Extrastriate Body Area (EBA) in the lateral occipito-temporal cortex and Fusiform Body Area (FBA) in the fusiform gyrus [54]). The activation of these areas has been interpreted as reflecting a dedicated neural circuitry for faces (“*face-selective hypothesis*” [21]), or a greater expertise in discriminating faces compared with other kinds of stimuli (“*expertise hypothesis*” [55]). The latter hypothesis found support in the FFA activation in participants trained to identify novel artificial objects sharing some typical constraints of faces (i.e., greebles; [56]), but subsequent studies reinterpreted this evidence in terms of subjects coding these stimuli as face-related [57].

While the OFA and EBA appear to underpin the neural representation of parts of faces and bodies, respectively, the FFA and FBA seem to reflect more holistic representations of these stimuli, i.e., processing the configurations of face- and body-parts into wholes [58]. Alongside the proximity of FFA and FBA in the posterior fusiform gyrus, the latter evidence raises the possibility that their functional integration underpins the ability to identify other individuals based on cues from both faces and bodies, particularly when a single cue-type is not sufficient for recognition [54]. This proposal fits with the notion that, among distinct neural pathways originating from these areas, a “ventral” pathway, running along the temporal cortex, underpins the semantic representation of specific concepts, i.e., the* identity* of familiar or unique stimuli. In particular, the polar sectors of the temporal and medial temporal cortex seem to be associated with the processing of unique houses or persons (e.g., the White House or President Obama) [59]. Along this pathway, single-cell recordings during awake-surgery have highlighted, in the human temporal and hippocampal cortex, neurons showing invariant responses to single persons, landmarks, and object [60]. The fact that these neurons are activated by different pictures of a same stimulus and some of them even by letter strings reporting its name strongly suggests their role in coding an abstract representation of specific concepts.

Another neural pathway of social perception involves the posterior portion of the lateral temporal cortex, where a hierarchical organization includes brain areas responding to pure motion (area MT/V5 in the inferior/middle temporal cortex), the typical motion of objects (middle temporal cortex), and biological motion (posterior portion of superior temporal sulcus; pSTS) [61] (Figure 2(a)). The pSTS represents a crucial hub of the brain network of social perception, processing the changeable features of biological stimuli and particularly their action-related motion patterns [62]. Neurophysiological studies have highlighted, in this region, single neurons responding to the observation of movements performed by different biological effectors, including eye-gaze [63]. Some of these neurons respond to complex visual patterns, such as the interaction between effector and objects, or a reaching action but only if the agent's gaze is directed to the target object [64]. Overall, the available evidence suggests that the pSTS plays a key role in the sensory binding of different features of biological motion, likely generating a superordinate representation of perceived actions [65]. Since pSTS neurons do not discharge during active movements, this region of the monkey brain does not display a “mirror-like” response. However, both neurophysiological data from the monkey [62] and neuroimaging evidence in human subjects [66, 67] suggest that the pSTS sends higher-level perceptual inputs to the frontoparietal mirror system associated with the analysis of the meaning of others' actions (see Section 2.2.1 and Figure 2(b)).

The pSTS is also part of another network, including the amygdala and orbitofrontal cortex (Amaral et al., 1992), associated with the processing of the affective value of observed stimuli. The amygdala is a key node of the social brain (Brothers, 1990), in which neuroimaging studies are associated with the emotional facets of social perception, such as the processing of facial expressions (Todorov et al., 2012) and judgments of trustworthiness [68]. This correlational evidence found support in lesional data showing the consequences of its damage, or abnormal functioning, on social cognitive processing [69] and real social interactions [70].

In line with the recent emphasis on the notion of “connectome” [71], diffusion imaging studies have started to address the structural connections underpinning the different facets of social cognition [72]. In the case of face processing, converging evidence shows the involvement of the inferior longitudinal fasciculus (IFL) and inferior frontooccipital fasciculus (IFOF), projecting from the occipital cortex to the anterior temporal and frontal cortex, respectively [73]. Their crucial role in connecting the nodes of the network subserving face processing is shown by studies relating distinct metrics of structural connectivity to face perception skills in normal conditions [74], physiological aging (disruption of the right IFL [75]), and in association with face blindness in developmental prosopoagnosia (disruption of both the right IFL and IFOF [76]). Preliminary evidence additionally shows the involvement of the superior longitudinal fasciculus (SLF), connecting temporal, parietal, and frontal regions [77] and particularly face-responsive portions of the STS with orbitofrontal and inferior frontal cortex ([78, 79].

### 2.2. 

#### 2.2.1. Social Understanding: Representing Others' Behavior

Since others' behavior is not completely predictable, the success of social interactions depends on the ability to decode their mental and, particularly, intentional states [80]. Interpreting others' behavior in terms of mental states, such as beliefs, desires, intentions, goals, experiences, sensations, and emotions, is thus a critical step for predicting their future actions [81]. This natural disposition to mentalizing entails the development of a “Theory of Mind” (ToM) based on the awareness that people have mental states, information, and motivations that may differ from one's own (Frith and Frith, 2006) [82]. On this assumption, mentalizing performance is typically measured with tasks assessing whether an individual is able to represent mental states, attributes them to oneself vs. other persons, and then, based on such attribution, correctly understands and/or predicts others' behavior [83–85].

**Figure 1:**
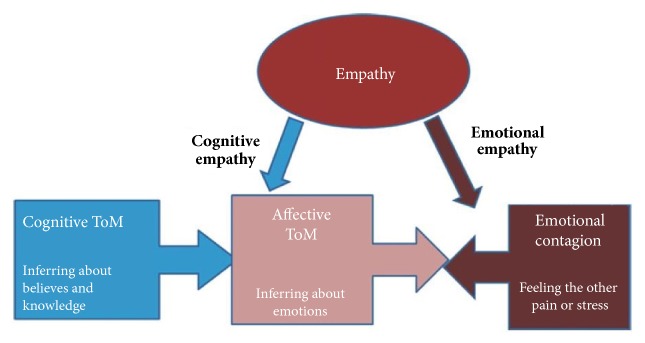
Empathy and mentalizing. The figure depicts Shamay-Tsoori et al.'s [91] model of the relationship between the key processes of social understanding, i.e., empathy and mentalizing. According to the model, cognitive mentalizing is a prerequisite for affective mentalizing, which however interacts with emotional empathy. Reproduced with permission from Shamay-Tsoori, Harari, Aharon-Peretz, and Levckovitz, [91].

Far from being a unique process, mentalizing involves several components and the integration of different facets of social understanding [86, 87]. Neuroimaging studies are providing increasing knowledge on the neural correlates of such components. A first crucial distinction regards the ability to attribute mental states vs. affective states, i.e.,* cold *or* cognitive ToM *vs.* hot or affective ToM*, respectively [88]. Moreover, representing others' thoughts, desires, feelings, and traits, i.e., mentalizing, differs from grasping and automatically sharing affective states, i.e., empathy [89]. On the other hand, these constructs are partially overlapping [90], and an influential model considers cognitive ToM a prerequisite for affective ToM, which additionally requires empathic skills ([91] see Figure 1).

In addition, a dissociation has been proposed between implicit and explicit mentalizing [80]: while the former would be present even in infants, who can ascribe false beliefs to agents from nonverbal behavior [2], explicit mentalizing represents a cognitively demanding sociocultural skill acquired by verbal instructions. Considerable evidence nowadays shows that explicit mentalizing develops slowly in the childhood [87]. Finally, based on computational complexity it is common to distinguish between first and higher-order Theory of Mind processing. First-order ToM, involving the representation of another individual's mental states (inclusive of both its affective and cognitive components) [92], develops between the age of 4 and 5 [93]. Second-order ToM, i.e., mentalizing what someone else is thinking or feeling about a third person's mental states [94], typically develops at the age of 6.

Social perception and in particular emotion decoding are considered to precede mentalizing [85]. The former stage would indeed reflect low-level perceptual processes providing inputs to the higher-level integrative and inferential processes associated with mentalizing [95]. On the other hand, mentalizing can influence social perception via top-down mechanisms mediated by long-term knowledge. This bidirectional relationship represents a core element of the influential Mindreading model [96], in which social perception and mentalizing represent different components of a larger system subserving the ability to perceive and respond appropriately to others' emotions and intentions [97]. This model entails three key perceptual detectors for mental states, gaze, and affective states, alongside a shared attention mechanism supporting the ability to selectively focus on specific stimuli and integrating the outcome of detector-specific basic perceptual processes. On top of this hierarchy, an advanced mentalizing ability allows us to perceive and respond appropriately to others' emotions, beliefs, and behaviors.

The kind of processes underpinning the mentalizing ability is, however, strongly debated (Goldman and Sripada, 2005). According to so-called “Theory-theory”, people act as naïve social scientists, developing psychological theories to infer others' mental states [98]. Based on the aforementioned mirroring process, “Simulation theory” rather states that we attribute mental states to others by simulating them in our own mind [45, 99]. A considerable literature, mostly based on neuroimaging data, suggests that different processes, revolving around simulative mechanisms vs. inferential routines, are recruited depending on the type of stimuli (visual vs. verbal) and instructions (implicit vs. explicit) ([66, 100] see [101]).

An alternative to both these approaches is represented by so-called “interaction theory” [102], stressing the role played by embodiment and direct perception when experiencing real social interactions (Froese and Gallagher, 2012). Based on the uniqueness of social interaction, in terms of the richness of incoming information and complexity of the responses, the advocates of this perspective aim to address social cognition from an interactor's point of view [103], also with innovative experimental designs grounded in virtual reality [104, 105], to investigate the mechanisms whereby individuals modulate their actions online [106]. This change of perspective involves shifting from “open-loop” to “closed-loop” scenarios where interactors influence one another dynamically, reciprocally, and continuously [107]. Neuroimaging studies based on this approach have shown that compared with the mere observation of social stimuli, being actively engaged in a social interaction activates a more extensive network of areas associated with perception-action coupling and affective evaluations, promoting motor responses coherent with the social stimulus [107]. These results highlight the potential implications of such an ecological approach not only for studying the neural bases of social cognition in normal individuals, but also for characterizing related disorders in pathological populations and for rehabilitation after brain damage. For example, recent evidence based on human-avatar online interactions shows that apraxics' motor impairments in a social reach-to-grasp task are abolished when patients are asked to interact with a virtual partner rather than performing actions on their own [108].

#### 2.2.2. Neural Correlates of Social Understanding

Distinct research lines, within social cognitive neuroscience, have addressed the neural bases of the ability to understand others' behaviors and decode their intentions and feelings. Most of the related evidence revolves around the mirror and mentalizing brain networks which, based on inputs from the pSTS (see Section 2.1.2), appear to underpin distinct levels of the hierarchy of social understanding [66, 109].

**Figure 2:**
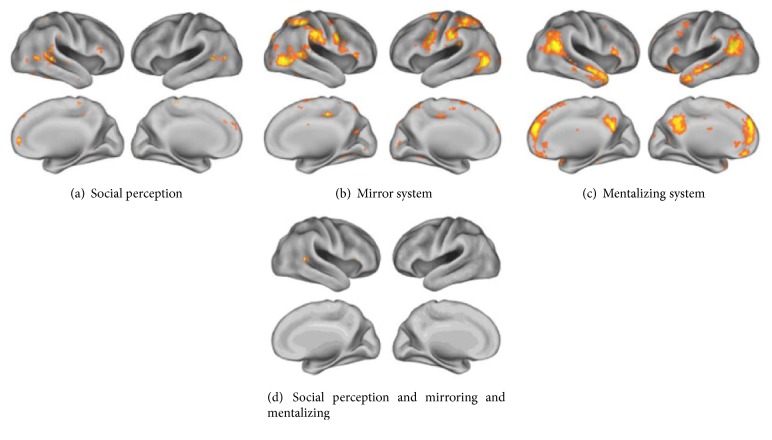
Brain networks of social cognition. Meta-analytic evidence for the neural networks underlying social perception (a), action observation (mirror system) (b), and mentalizing (Theory of Mind system) (c). As shown in the bottom sector of the figure, these three networks overlap in the STS, a crucial hub of the social brain providing inputs to both the mirror and mentalizing systems [66]. Reproduced with permission from Yang, Rosenblau, Keifer, and Pelphrey,* An Integrative Neural Model of Social Perception, Action Observation, and Theory of Mind*, Neuroscience and Biobehavioral Reviews, 51 (2015) 263–275, doi:10.1016/j.neubiorev.2015.01.020.

The mirror system includes inferior frontal, premotor, and parietal regions which are activated both when performing an action and when observing the same action performed by someone else [41] (Figure 2(b)). This network is considered to underpin a variety of action-related social functions, from action recognition [110] and imitation learning (Vogt et al., 2007) to the context-based decoding of so-called “private goals,” e.g., grasping a cup to drink vs. to clean the table (Iacoboni et al., 2005). The mirror system is anatomically and functionally distinct from the mentalizing system, which includes the medial prefrontal cortex (mPFC), temporoparietal junction (TPJ), medial precuneus/posterior cingulate cortex, and temporal poles [86, 111, 112] (Figure 2(c)). This network of areas is typically engaged when others' intentions cannot be automatically derived from visual cues and must thus be inferred in terms of thoughts and beliefs [101, 109].

Therefore, a superordinate dimension eliciting the specific recruitment of the mirror vs. mentalizing systems is represented by the aim to identify, respectively,* how* (executed movements associated with a behavioral state) vs.* why* (beliefs and intentions associated with a mental state) an action is performed [113–115]. The mirror and mentalizing systems seem thus to play complementary roles in processing others' intentions, driven by the presence of, respectively, biological actions vs. abstract information (e.g., observing real scenes vs. reading stories) or implicit vs. explicit instructions (e.g., to passive observe vs. to infer characters' intentions) [101], and by identifying* how* vs.* why* the character is expressing a feeling (i.e., explicit identification vs. attribution [114]).

While the evidence reviewed above involves the attribution of intentions and cognitive states, other research lines have addressed the neural bases of empathy, i.e., grasping others' feelings through their direct resonance in the observer's brain. This process seems to recruit a mirror-like mechanism specific for different kinds of empathic responses, involving the same brain regions associated with their first-person experience rather than the frontoparietal mirror network. This is the main finding of a series of studies which have reported the involvement of (a subset of) the same brain regions when directly experiencing, and when attending in someone else, specific affective or sensory stimulations. Such a mechanism has been described for the direct and vicarious experience of pain (anterior insula and anterior cingulate cortex, i.e., the affective sector of the so-called pain matrix [116, 117]), disgust (anterior insula [118]), tactile sensations (secondary somatosensory cortex SII [119]), and even regret for the outcomes of choices (orbitofrontal cortex and anterior cingulate cortex [120, 121]). In keeping with the notion of “mirroring,” these results suggest that the observation, or even the mere awareness [116, 117], of another person in a particular emotional state may automatically activate the neural representation of the same state in the observer. Such representation includes its associated autonomic and somatic responses, neurally associated with the activation of the anterior insula and dorsal anterior cingulate cortex [122, 123], which provides support to the concept of a mirroring, sensorimotor, and nature of empathy [124]. This notion is strengthened by recent evidence on the neurophysiological correlates of facial mimicry, i.e., the unconscious and unintentional automatic response to the facial expressions of others [125]. The simultaneous recording of facial muscular reactivity (via electromyography, EMG) and brain activity (via* f*MRI) highlighted a correlation between spontaneous facial muscle reactions to facial expressions and brain activity in the frontoinsular and inferior parietal “mirror” sectors associated with their motor simulation. Overall, considerable evidence indicates that such a limbic, visceromotor, mirroring system for shared sensory and emotional experience provides the neural framework for emotional insights into other minds.

Both mirroring and mentalizing have been associated with structural connections between temporal, parietal, and frontal lobes underpinned by the superior longitudinal fasciculus [72]. The latter has been indeed associated with individual differences in abilities such as emotion recognition [126, 127], empathy [128], and imitation [129]. Other facets of embodied cognition have been ascribed to further limbic tracts, i.e., the uncinate fasciculus linking medial temporal and orbitofrontal cortex [130], involved in socioemotional processing [131], and the anterior thalamic radiation connecting the hypothalamus and limbic structures to prefrontal and anterior cingulate cortex [132], associated with affective processing and emotional regulation [133]. In keeping with their role in face processing, the inferior longitudinal fasciculus (ILF) and inferior frontooccipital fasciculus (IFOF) have been also associated with emotion recognition and empathy skills both in healthy [128, 134] and brain-lesioned [135, 136] individuals. In addition to the SLF, mentalizing seems to be supported also by the cingulum (linking medial prefrontal, posterior cingulate and medial temporal cortex [130]) and arcuate fasciculus (connecting the temporoparietal junction with prefrontal cortex [137]). Mentalizing abilities have been related to the degree of axonal injury in the left cingulum in brain-lesioned children [138] and in the arcuate fasciculus, near the temporoparietal junction, in high-functioning autistic individuals [139]. Strong evidence for this association comes also from direct electrical stimulation during neurosurgery, showing that the virtual disconnection of these tracts results in a marked decrease of mentalizing performance [140, 141].

### 2.3. 

#### 2.3.1. Social Decision-Making

Understanding others' behaviors in terms of dispositions and intentions is often critical for making appropriate decisions in a variety of social contexts. Most choices are made within direct or indirect social interactions within complex and dynamic environments. They will thus depend either on the choices already made by others (if they are known) or on our prediction of the choices they will make (if concurrent with our own ones) and more generally on the awareness of their consequences for both ourselves and others [142]. From the economic standpoint, studying decisions made in different types of social context, or even within social interactions, is aimed at identifying the optimal choice among the available ones. On the other hand, psychological studies have shown several examples of preferences which seem to reflect prosocial and/or affective considerations even more than economic utilities. Researchers have thus begun to investigate the social and cognitive variables modulating social decision-making using tasks originally developed in distinct research fields within the economic sciences.

One typical example is represented by studies modeling agents' choices with the tools of Game Theory. The latter is based on rigorous models aiming to identify the optimal choice for interacting agents, in different possible situations in which they know the respective outcomes of each possible choice and they can, or cannot, make agreements before choosing. As anticipated, however, real human choices often deviate from the predictions of such models. For instance, classical Game Theory predicts that a group of rational players will make decisions to reach outcomes, known as Nash equilibria [143], from which no player can increase his/her own playoff unilaterally. Still, considerable evidence shows that people introduce psychological and prosocial considerations in their strategies, which appear to be less selfish and more fairness-oriented than predicted by economic models [144]. Typical examples of such prosocial attitude are represented by the usual response patterns observed in three tasks entailing two interacting players, popularly known as Ultimatum, Dictator, and Trust games (Fehr and Fischbacher, 2006).

In the* Ultimatum Game* [145], the proposer is asked how much of a financial endowment she/he is willing to send to an unknown responder. The latter can accept or reject the offer: in the first case, the sum is divided as proposed; in case of rejection, instead, no one receives anything. Against the economic prescription, i.e., to accept any offer as a responder and thus to offer as less as possible as a proposer [146], people usually propose “fair” offers [147] and reject unfair offers [148], although with some cultural differences [149], rejection-rates increase substantially as offers decrease in magnitude. A similar trend is found in the* Dictator game*, although the responder can only accept the proposer's offer.

In the* Trust Game*, two players receive the same initial endowment. Then, the “trustor” player decides how much of this sum to send to a trustee. Both players know that the transferred amount will be multiplied by a factor >1. The trustee must then decide whether to return some of her/his payoff to the trustor. If she/he honors trust, both players end up with a net monetary increase. If instead the trustee keeps the entire amount, the trustor ends up with a loss. In the case of a single interaction (i.e., “one-shot”), a rational and selfish trustee would not be expected to honor the first player's trust. Therefore, the latter should never trust the other player. Against this prediction, instead, in most studies the first player sends some money to the second one, with trust being typically reciprocated [150].

Both in their “one-shot” and iterated versions, these tasks typically highlight the willingness to punish, at own expenses, defectors who will never be met again [144, 151]. Considerable evidence seems indeed to show the role played, in real human interactions, by an* expectation of reciprocity*. The latter is the basis of the “tit-for-tat” strategy, i.e., trusting the partner at the first move and then replicating her/his next moves, in which both informatic simulations and psychological studies highlight as the natural strategy in social interactions (Axelrod and Hamilton, 1981). Importantly, this strategy requires the identification and punishment of defectors, even when this is not directly beneficial to the punisher. Since the simple presence vs. absence of the possibility to punish has been shown to increase vs. reduce cooperation in social interaction [151], this behavior has been called “altruistic punishment” because its costs will benefit individuals other than the punisher. While representing another puzzling behavior for economic theories, real interaction-games have shown that altruistic punishment is a key prerequisite for cooperative behavior to spread in a society [151]. There must exist, then, some incentive to behaviors which are socially advantageous, but individually expensive. A possible incentive for altruistic punishment by single individuals has been found in the strong negative emotions associated with unfairness, defection, and abuse of one's own trust, eliciting a “desire of revenge” [151]. In simpler words, anticipating the pleasure inherent in satisfying such desire would represent the incentive to punishment behaviors which, although irrational in purely economic terms for the single individual, exert prosocial consequences at the society level.

While classical economic models had largely ignored the influence of emotions on decision-making, growing evidence at the crossroad between cognitive neuroscience and economics is showing the effect of affective processing on actual choices [152]. It is now widely acknowledged that decision-making is driven by anticipated outcome-related feelings and particularly by the attempt to experience positive feelings associated with gains and prosociality and to avoid negative feelings such as disappointment for a loss, regret for a foregone outcome, or guilt for the adverse consequences of one's choices for another [153]. The neural bases of these processes constitute the subject of neuroeconomics, a lively research field at the crossroad among neural, psychological, and social sciences.

#### 2.3.2. Neural Correlates of Social Decision-Making

Understanding others' affective and cognitive states and particularly intentions is often a crucial step for different facets of social decision-making. These might include anticipating others' choices in a strategic context, or planning the reaction to another's defection, e.g., an unfair proposal in the Ultimatum Game, or unreciprocated trust in the Trust Game. While the aforementioned psychological studies have highlighted actual behaviors inconsistent with “rational” economic predictions, neuroscientific data suggest that the typical human prosocial attitude might largely reflect motivational drives associated with brain regions underlying affective and hedonic evaluations. This research field is indeed grounded in the notion that the weight of affective drives, largely acknowledged in individual decision-making (e.g., [120, 121, 154, 155]), is even enhanced when making choices in a social context [156, 157].

A fast-growing literature is unveiling a mosaic of brain regions underlying the different facets of this process. First, the activation of the anterior insula in association with the receipt and rejection of unequal offers by another human subject [158] might reflect the negative affective reactions elicited by unfairness. Interestingly, accepting unfair offers reflects in increased activity of the dorsolateral prefrontal cortex, a key node of the executive network associated with cognitive control and response inhibition. The latter evidence has been initially interpreted in terms of the role played by this region in inhibiting the negative affects prompting the rejection of unfair offers [158]. However, against this hypothesis further studies have shown an increase of acceptance rate after its deactivation with transcranial magnetic stimulation (TMS) [159, 160]. The dorsolateral prefrontal cortex might thus underpin the selfish drive to accept every offer, rather than the prosocial aptitude toward altruistic punishment. On the other hand, the fact that punishing defectors reflects in the activation of the ventral striatum [161], the key node of the brain reward network (Schultz et al., 2006), suggests that altruistic punishment might be also stimulated by the rewarding experience implicit in satisfying the desire for revenge against nonreciprocators. Due to its costs, such behavior requires to weigh economic and hedonic outcomes, a tradeoff involving the ventromedial prefrontal cortex [161]. Overall, the activation of the striatum in association with “tit-for-tat” behaviors and particularly with reciprocated cooperation [162] highlights a neurobiological interpretation of the, economically irrational, tendency to prefer prosocial behaviors over individual gratifications [163]: the subjective utility associated with mutual cooperation would represent a short-term social reward outweighing that resulting from unilateral defection (which, in contrast, might additionally reflect in negative feelings such as shame and guilt).

While these data seem to highlight a natural human disposition to prosocial behavior and sharing of resources, less optimistic evidence comes from studies investigating the neural bases of altruism and charity, i.e., costly behaviors providing benefits only to other people. On the one hand, the activation of the ventral striatal hub of the reward network [164, 165] might suggest that altruistic behavior is rewarding in itself, which could be interpreted as an evidence against the existence of “pure” altruism. Moreover, other studies have shown, in the same subjects, overlapping ventral striatal activations when deciding to donate money while knowing to be observed and when deciding to keep the money while knowing that no one was observing them [166]. These results suggest the opportunity to reframe the theoretical speculations and empirical analyses of the putative human prosocial, or even altruistic, disposition in a broader perspective merging economic, psychological and neuroscientific evidence.

## 3. Age-Related Changes in Social Cognition

A growing literature on age-related changes in cognitive proficiency reveals that physiological aging entails both losses and gains of functions (Kensinger et al., 2017) [167]. Despite a global decrease of cognitive efficiency, some facets of social cognitive and affective processing remain stable or even improve with age [168], bringing potential benefits to everyday functioning [169].

Such changes involve the interaction of multiple processes, i.e., disruption of functions, resource limitations, and reallocation, as well as compensative mechanisms (Kensinger et al., 2017). In turn, these processes are modulated by a wide range of factors including, among others, individual differences in education [170], level of fluid cognition [171], and resource availability [172]. Within this complex scenario, two variables are considered to provide the strongest contribution to age-related changes in social cognition [173].

The first variable concerns the cooperation vs. competition between automatic and controlled processes. Since aging mainly impacts executive control (von Hippel and Henry 2012), a significant reduction of the ability to inhibit automatic responses can result in socially disinhibited and inappropriate behaviors [174]. The same mechanism appears to facilitate stereotypical thoughts, which are automatically activated in the presence of stereotyped group members, making older adults more inclined to show prejudices than younger adults (von Hippel and Henry 2012).

Secondly, changes in social cognition seem to depend on whether and to what extent tasks rely on novel information processing vs. accumulated experience [175]. Despite a global decrease of cognitive efficiency (in terms of speed processing, memory, complex reasoning, attention, and inhibitory control), as well as physical [176] and perceptual [177] functioning, several studies reported smaller age-related effects in domains related to past experience, including vocabulary and general knowledge [175]. This is a crucial notion, since these skills might contribute to specific facets of social cognitive and affective processing and thus partially compensate the overall cognitive decline.

For instance, although older adults perform worse than young adults on memory recall tasks, even when presented with social and affective stimuli [178], they show equally, or even more, effective emotion regulation skills [171]. While the latter evidence may appear at odds with an executive decline, emotion regulation may require less resources in older than young adults due to the improved procedural knowledge accumulated throughout life [168]. In addition, older adults may allocate a greater proportion of resources to emotion regulation compared to younger adults [179], due both to the possible prioritization of arousing and to self-relevant information (Kensinger et al., 2017), and to age-related motivational changes toward the maximization of the emotional satisfaction in the “here and now” [168].

Overall, these findings highlight the complexity of age-related changes in social cognition, which are deeply intertwined with both the physiological decrease of cognitive functioning and the shifts in life goals. We will briefly review the available evidence on the changes reported in the three domains of social cognition previously described.

### 3.1. Age-Related Changes in Social Perception

As discussed in Section 2.1.1, faces represent a crucial source of social signals, and emotion recognition from facial expressions is a critical prerequisite for appropriate interpersonal communication and social functioning [180] (von Hippel and Henry, 2012).

While aging is associated with significantly decreased performance in recognizing negative emotions such as fear, sadness, and anger [180], older adults perform better than younger ones in the case of positive emotions (i.e., happiness and surprise) and disgust. This evidence has been ascribed to the* top-bottom bias*, indicating age-related changes in face-processing strategies: older, compared with younger, adults are more likely to focus on the bottom half of the face (mouth or nose), which provides information concerning the muscular contractions specifically associated with happiness and disgust [181], rather than on the eyes [182]. This pattern might reflect functional and/or structural age-related changes within the face-processing brain network—including the STS, medial PFC and amygdala—associated with eye-gaze perception and decoding [167].

On the other hand, the decline in recognizing negative emotions from faces might be also attributed to the “*age-related positivity effect”* [183], indicating the older adults' tendency to focus more on positive than negative stimuli compared with younger adults. This effect, consistently described also in attention ad memory domains [184], has been linked to age-related changes in emotion regulation mechanisms, helping elders to preserve a better mood [185]. These changes might reflect the fact that, in the elderly, only negative stimuli are associated with the activation of the medial prefrontal cortex (PFC), possibly supporting top-down emotion regulation processes aimed to inhibit negative emotions [186].

### 3.2. Age-Related Changes in Social Understanding

The preservation of functions underlying social understanding, such as emotional sharing and the attribution of cognitive or affective states to others, predicts successful outcomes in aging [187]. In the attempt to disentangle specific changes in the different facets of social understanding, several studies have shown a prominent age-related decline in its* cognitive* components (i.e., mentalizing and social metacognition), with a relative conservation, or even an enhancement, of the* affective* ones (i.e., empathy and compassion) [167, 173, 188]. Also in this case, the former evidence may reflect an overall decline in executive control and fluid intelligence [189], associated with reduced activity of the dorsolateral PFC [167]. Additionally, older adults seem to shift their motivations: according to the* socioemotional selectivity theory* they disengage their focus from future-oriented goals and prioritize social and emotional meaningful activities by selectively allocating more resources on emotional processing and emotion regulation strategies [190]. This view is supported by a study reporting age-related neurostructural changes in 883 healthy individuals. While cortical thickness decreased with age in brain regions related to executive functioning, such as the dorsal ACC alongside the superior and lateral sectors of the PFC, no age affect was found in regions typically engaged in emotion regulation, such as the ventromedial PFC and ventral ACC [191].

### 3.3. Age-Related Changes in Social Decision-Making

Alongside an enhancement of affective processing skills, different facets of social behavior and decision-making, like generativity and prosociality, undergo substantial changes with age.

Generativity, i.e., the tendency to expand the individual focus of concern beyond oneself [192], becomes a prominent challenge in late life, prompting the desire of cooperation between generations and the need for older adults to offer emotional support and mediate conflicts, which are perceived as essential goals for survival (Gurven and Kaplan, 2009). Compared with young people, older adults endorse more generative goals and other-focused problem solving [193]. Moreover, both the feeling of pity and the willingness to help others seem to progressively increase with age [194].

Closely related to social affective skills, also the tendency to prosociality seems to increase in late life [195]. In line with the* socioemotional selectivity theory*, contexts relevant to social and affective goals might motivate older adults, even more than younger ones, to help others, since empathy and/or compassion represent powerful skills capable of promoting prosocial behaviors [195]. This is the core of “empathic concern” [196], whereby acting to benefit needy others can mitigate the negative emotional arousal induced by experiencing their needs. The enhancement of emotion regulation skills might thus mediate the higher prosociality displayed by older adults, ultimately increasing their well-being, satisfaction and emotional fulfillment [193].

## 4. Altered Social Cognition in Neurodegenerative Diseases

Increasing evidence highlights a variety of social cognitive impairments in different neurological (e.g., neurodegenerative diseases, traumatic brain injuries, and brain tumors) and psychiatric (e.g., mood disorders, autism, and schizophrenia) conditions [197–200]. These alterations are mainly associated with the functional consequences of neuropathological processes or brain lesions affecting regions and networks underlying social cognition skills.

Within the realm of neurodegenerative diseases, pathological changes in social cognition and behavior are a major hallmark of the frontotemporal dementia (FTD) disease spectrum, including the primary progressive aphasias (i.e., semantic, nonfluent, and logopenic variants) [201] and the behavioral variant (bvFTD) [202]. Due to the progressive degeneration of frontobasal and limbic networks associated with the processing of emotional and social cues [203–207], bvFTD represents a prototypical example of the breakdown of social cognition. A marked neurocognitive impairment has been reported, in this disease, in all the domains previously discussed, from emotion recognition and social understanding to judgment involving social dilemmas and violations (Elamin et al., 2013). Despite similar deficits in emotion recognition and social understanding [208, 209], bvFTD and both the semantic and the nonfluent FTD variants have been associated with different patterns of structural damage within a frontoinsular-temporal network which is also known as “social context network” [210]. This model is based on the notion that different social cognitive processes are encapsulated into specific context circumstances, having an intrinsic social meaning. Specific patterns of social cognitive impairment, typical of neurological and psychiatric diseases, might thus arise from selective dysfunctions within a distributed network causing a global impairment in the processing of social context information. This network is considered to include three main hubs with specific functions, i.e., (1) frontal areas, supporting the updating of context cues to make predictions; (2) temporal cortex, underlying the consolidation of value-based learning of contextual associations; (3) insular cortex, managing the convergence between emotional and cognitive states related to the coordination between external and internal milieus and thus facilitating frontotemporal interactions in processing social contexts.

Further cues into the abnormal social brain come from the literature revolving around the FTD-Amyotrophic Lateral Sclerosis (ALS) continuum hypothesis [211]. The growing evidence on the neuropathological, genetic, neuroimaging, and clinical commonalities between the two conditions [212–217] now includes social cognitive deficits, which have been revealed also in ALS patient without dementia [218].

Social cognitive impairments have been described also in other neurodegenerative disorders. Although these symptoms are not considered central or typical expressions of these diseases, the impairment can involve one or more of the domains reviewed before. As discussed in the next paragraphs, social perception and social understanding are, to date, the most frequently investigated domains in neurodegenerative disorders.

### 4.1. Altered Social Perception in Neurodegenerative Diseases

With respect to social perception, evidence exists for abnormal visual and/or auditory (i.e., based on prosodic cues) recognition of basic emotions, especially involving negative emotions, in bvFTD [219–222]. Interestingly, emotion recognition from faces discriminates bvFTD from other neurodegenerative, as well as psychiatric, diseases [207]. However, abnormal affective processing and emotion recognition (particularly for negative emotions) have been found also in other disorders (Elamin et al., 2013) [223], such as ALS [126, 224], Parkinson's disease [225], corticobasal syndrome and progressive supranuclear palsy [226], and Huntington's disease [223, 227], as well as Alzheimer's disease (AD) and mild cognitive impairment, particularly when subtle or static emotional stimuli are presented [8, 228].

### 4.2. Altered Social Understanding in Neurodegenerative Diseases

The studies addressing social understanding in neurodegenerative diseases are contributing to unveil a complex scenario, with different disorders reflecting in distinct patterns of functional impairment. Defective mentalizing skills have been reported in bvFTD and AD [229]. However, while in AD this deficit likely reflects a global cognitive breakdown, bvFTD patients display a relatively selective impairment in affective mentalizing [229], likely reflecting their marked difficulties with empathic abilities [230]. In line with the continuum hypothesis, this pattern has been also described in a subset of ASL patients displaying a prominent impairment in the processing of emotional cues (Cerami et al., 2013) [127]. In Parkinson's disease, early mentalizing deficits are accompanied by decreased empathic skills in the later disease stages, reflecting the progression of the pathology from the dorsolateral prefrontal to orbitofrontal circuits (Elamin et al., 2013). In Huntington's disease, the impairment of both cognitive and affective components of social understanding is often associated with the severity of executive decline and motor symptoms [223].

### 4.3. Altered Social Decision-Making in Neurodegenerative Diseases

Abnormal performance in tasks assessing individual decision-making has been described in different neurodegenerative diseases, such as FTD, AD, Parkinson's disease, and Huntington disease (see [231] for a review). Instead, the evidence on social decision-making in neurodegenerative diseases is still limited and mainly related to bvFTD and AD [223]. In particular, bvFTD patients display a significant reduction in the tendency to prosociality [232] and cooperative behavior [233] (O'Callagan et al., 2015). In line with the “social context network” model described above [210], such changes might reflect the damage in frontostriatal areas supporting the generation and update of predictions based on social contextual information.

## 5. Conclusions

The data reviewed here summarize the main results of social cognitive neuroscience in the attempt to unveil the brain networks underlying the humans' automatic disposition to make sense of others' behavior. While most of the initial efforts within this lively research field dealt with the “social brain” in healthy individuals, its most recent developments are concerned with identifying the changes associated with physiological aging or different pathological conditions. A growing literature shows that the multilevel approach of social cognitive neuroscience, connecting seemingly distinct drivers of human behavior such as hormones or prosocial motivations [234], constitutes a platform providing experimental paradigms for targeting specific social cognitive processes, as well as objective metrics for assessing their impairment, or the effectiveness of remediation procedures, in different neuropsychiatric diseases [7].

The advancements in parcellating social cognitive processes and their neural bases nowadays allow design interventions based on robust evidence at the level of the construct of interest (e.g., face processing), or of deeper neurobiological mechanisms such as the modulation of amygdala activity by oxytocin (Ebert and Brune, 2017). The complexity of social cognition and its multifaceted nature indeed reflect in the variety of different remediation procedures which have been already proposed to improve social skills and to assess their impact beyond the trained process. Different approaches aim to improve either basic cognitive skills, to increase relational competence via training strategies underlying the analysis of social context and emotional information (i.e., “wide interventions; Peyroux and Frank, 2014), or specific components of social cognition such as emotion recognition [235], mentalizing [236], or empathy (Klimecki et al., 2013) (i.e., “targeted interventions”), particularly in schizophrenia [237] and autism [238, 239]. Meta-analytic results highlight moderate training effects on emotion recognition and mentalizing, with such improvements being transferred to daily social life [240], but also limited success in remediating more complex, higher-order social cognitive functions [241]. Possible explanations for this negative evidence might include the lack of consideration of basic cognitive impairments and of real-world social situations characterized by a basic property of social cognition such as the mutual interdependence between agents. As previously discussed (Section 2.2.1), the potential implications of novels paradigms entailing real or virtual social interactions represent one of the most promising challenges for social neuroscience [106], already supported by positive outcomes in neurological patients [108].

More generally, the available evidence suggests that the effectiveness of social cognitive remediation depends on “baseline” skills and that successful programs require adapting management strategies based on individual profiles. A detailed description of social cognitive processes and their neural correlates is thus critical to tailor remediation protocols to target specific brain networks and their associated cognitive functions. By summarizing the extensive available evidence on the neural bases of social cognition, the present review highlights specific domains which should be evaluated in pathological populations, taken into consideration when designing novel tests [242, 243] or rehabilitation procedures [244], and addressed in original studies. As in all areas of empirical research, the quality of the answers depends on the quality of the questions. This is one of the main reasons why the increasing interaction among social and clinical as well as basic and translational research areas represents one of the most exciting developments within cognitive neuroscience.

## References

[1] Frith C. D., Frith U. (2007). Social Cognition in Humans. *Current Biology*.

[2] Scott R. M., Baillargeon R. (2017). Early False-Belief Understanding. *Trends in Cognitive Sciences*.

[3] Krupenye C., Kano F., Hirata S., Call J., Tomasello M. (2016). Great apes anticipate that other individuals will act according to false beliefs. *Science*.

[4] Slaughter V., Imuta K., Peterson C., Henry J. (2014). Meta-analysis of theory of mind and peer popularity in the preschool and early school years. *Child Development*.

[5] Jones D. E., Greenberg M., Crowley M. (2015). Early social-emotional functioning and public health: The relationship between kindergarten social competence and future wellness. *American Journal of Public Health*.

[6] Holt-Lunstad J., Smith T. B., Layton J. B. (2010). Social relationships and mortality risk: a meta-analytic review. *PLoS Medicine*.

[7] Henry J. D., von Hippel W., Molenberghs P., Lee T., Sachdev P. S. (2016). Clinical assessment of social cognitive function in neurological disorders. *Nature Reviews Neurology*.

[8] Phillips L. H., Scott C., Henry J. D., Mowat D., Bell J. S. (2010). Emotion Perception in Alzheimer's Disease and Mood Disorder in Old Age. *Psychology and Aging*.

[9] Kennedy D. P., Adolphs R. (2012). The social brain in psychiatric and neurological disorders. *Trends in Cognitive Sciences*.

[10] Kawamichi H., Sugawara S. K., Hamano Y. H., Makita K., Kochiyama T., Sadato N. (2016). Increased frequency of social interaction is associated with enjoyment enhancement and reward system activation. *Scientific Reports*.

[11] Cacioppo S., Grippo A. J., London S., Goossens L., Cacioppo J. T. (2015). Loneliness: clinical Import and Interventions. *Perspectives on Psychological Science*.

[12] Layden E. A., Cacioppo J. T., Cacioppo S. (2017). Perceived social isolation is associated with altered functional connectivity in neural networks associated with tonic alertness and executive control. *NeuroImage*.

[13] Steptoe A., Shankar A., Demakakos P., Wardle J. (2013). Social isolation, loneliness, and all-cause mortality in older men and women. *Proceedings of the National Acadamy of Sciences of the United States of America*.

[14] Holwerda T. J., Deeg D. J. H., Beekman A. T. F. (2014). Feelings of loneliness, but not social isolation, predict dementia onset: Results from the Amsterdam Study of the Elderly (AMSTEL). *Journal of Neurology, Neurosurgery & Psychiatry*.

[15] Lieberman M. D. (2007). Social cognitive neuroscience: A review of core processes. *Annual Review of Psychology*.

[16] Malle B. F. (2005). Three Puzzles of Mindreading. *Other Minds: How Humans Bridge the Divide between Self and Others*.

[17] Frith C. D., Frith U. (2012). Mechanisms of social cognition. *Annual Review of Psychology*.

[18] Malle B. F. (2004). *How the Mind Explains Behavior: Folk Explanations, Meaning, and Social Interaction*.

[19] Vogeley K. (2017). Two social brains: neural mechanisms of intersubjectivity. *Philosophical Transactions of the Royal Society B: Biological Sciences*.

[20] Dolan R. J. (2002). Emotion, Cognition, and Behavior. *Science*.

[21] McKone E., Kanwisher N., Duchaine B. C. (2007). Can generic expertise explain special processing for faces?. *Trends in Cognitive Sciences*.

[22] Kato M., Konishi Y. (2013). Where and how infants look: The development of scan paths and fixations in face perception. *Infant Behavior & Development*.

[23] Adams R. B., Albohn D. N., Kveraga K. (2017). Social Vision: Applying a Social-Functional Approach to Face and Expression Perception. *Current Directions in Psychological Science*.

[24] Maurer D., Le Grand R., Mondloch C. J. (2002). The many faces of configural processing. *Trends in Cognitive Sciences*.

[25] Yovel G., Kanwisher N. (2004). Face perception: Domain specific, not process specific. *Neuron*.

[26] Adams R. B., Nelson J. A., Matsumoto H. C. H. D., Frank M. G. (2016). Eye Behavior and Gaze. *Apa Handbook of Nonverbal Communication*.

[27] Baron-Cohen S., Jolliffe T., Mortimore C., Robertson M. (1997). Another advanced test of theory of mind: Evidence from very high functioning adults with autism or Asperger syndrome. *Journal of Child Psychology and Psychiatry and Allied Disciplines*.

[28] Adolphs R. (2002). Recognizing emotion from facial expressions: psychological and neurological mechanisms. *Behavioral and Cognitive Neuroscience Reviews*.

[29] Santana S. E., Dobson S. D., Diogo R. (2014). Plain faces are more expressive: Comparative study of facial colour, mobility and musculature in primates. *Biology Letters*.

[30] Cole P. M., Martin S. E., Dennis T. A. (2004). Emotion regulation as a scientific construct: Methodological challenges and directions for child development research. *Child Development*.

[31] Ekman P., Friesen W. V., O'Sullivan M. (1987). Universals and cultural differences in the judgments of facial expressions of emotion. *Journal of Personality and Social Psychology*.

[32] Elfenbein H. A., Ambady N. (2002). On the universality and cultural specificity of emotion recognition: A meta-analysis. *Psychological Bulletin*.

[33] Dael N., Mortillaro M., Scherer K. R. (2012). Emotion expression in body action and posture. *Emotion*.

[34] Scherer K. R. (1995). Expression of emotion in voice and music. *Journal of Voice*.

[35] Atkinson A. P., Dittrich W. H., Gemmell A. J., Young A. W. (2004). Emotion perception from dynamic and static body expressions in point-light and full-light displays. *Perception*.

[36] Bachorowski J.-A. (1999). Vocal expression and perception of emotion. *Current Directions in Psychological Science*.

[37] Johnstone T., Scherer K. R., Lewis M., Haviland J. (2000). Vocal Communication of Emotions. *The Handbook of Emotion*.

[38] Sauter D. A., Eisner F., Ekman P., Scott S. K. (2010). Cross-cultural recognition of basic emotions through nonverbal emotional vocalizations. *Proceedings of the National Acadamy of Sciences of the United States of America*.

[39] Aucouturier J. J., Johansson P., Hall L., Segnini R., Mercadié L., Watanabe K. (2016). Covert digital manipulation of vocal emotion alter speakers’ emotional states in a congruent direction. *Proceedings of the National Acadamy of Sciences of the United States of America*.

[40] Hyniewska S., Sato W. (2015). Facial feedback affects valence judgments of dynamic and static emotional expressions. *Frontiers in Psychology*.

[41] Gallese V., Fadiga L., Fogassi L., Rizzolatti G. (1996). Action recognition in the premotor cortex. *Brain*.

[42] Rizzolatti G., Sinigaglia C. (2010). The functional role of the parieto-frontal mirror circuit: interpretations and misinterpretations. *Nature Reviews Neuroscience*.

[43] Adolphs R., Damasio H., Tranel D., Cooper G., Damasio A. R. (2000). A role for somatosensory cortices in the visual recognition of emotion as revealed by three-dimensional lesion mapping. *The Journal of Neuroscience*.

[44] Niedenthal P. M., Brauer M. (2012). Social functionality of human emotion. *Annual Review of Psychology*.

[45] Gallese V., Keysers C., Rizzolatti G. (2004). A unifying view of the basis of social cognition. *Trends in Cognitive Sciences*.

[46] Wood A., Rychlowska M., Korb S., Niedenthal P. (2016). Fashioning the Face: Sensorimotor Simulation Contributes to Facial Expression Recognition. *Trends in Cognitive Sciences*.

[47] Niedenthal P. M., Barsalou L. W., Winkielman P., Krauth-Gruber S., Ric F. (2005). Embodiment in attitudes, social perception, and emotion. *Personality and Social Psychology Review*.

[48] Anderson J. R. (1983). A spreading activation theory of memory. *Journal of Verbal Learning and Verbal Behavior*.

[49] Fodor J. A. (1975). *The Language of Thought*.

[50] McNamara T. P. (1992). Theories of Priming: I. Associative Distance and Lag. *Journal of Experimental Psychology: Learning, Memory, and Cognition*.

[51] Bower G. H. (1981). Mood and memory. *American Psychologist (Salma)*.

[52] Winkielman P., Niedenthal P. M., Oberman L. M., Pineda J. A. (2008). Embodied Perspective on Emotion-Cognition Interactions. *Mirror Neuron Systems. Contemporary Neuroscience*.

[53] Bernstein M., Erez Y., Blank I., Yovel G. (2018). An Integrated Neural Framework for Dynamic and Static Face Processing. *Scientific Reports*.

[54] Peelen M. V., Downing P. E. (2007). The neural basis of visual body perception. *Nature Reviews Neuroscience*.

[55] Diamond R., Carey S. (1986). Why Faces Are and Are Not Special. An Effect of Expertise. *Journal of Experimental Psychology: General*.

[56] Tarr M. J., Gauthier I. (2000). FFA: A flexible fusiform area for subordinate-level visual processing automatized by expertise. *Nature Neuroscience*.

[57] Brants M., Wagemans J., de Beeck H. P. O. (2011). Activation of fusiform face area by greebles is related to face similarity but not expertise. *Cognitive Neuroscience*.

[58] Taylor J. C., Wiggett A. J., Downing P. E. (2007). Functional MRI analysis of body and body part representations in the extrastriate and fusiform body areas. *Journal of Neurophysiology*.

[59] Simmons W. K., Martin A. (2009). The anterior temporal lobes and the functional architecture of semantic memory. *Journal of the International Neuropsychological Society*.

[60] Quiroga R. Q., Reddy L., Kreiman G., Koch C., Fried I. (2005). Invariant visual representation by single neurons in the human brain. *Nature*.

[61] Beauchamp M. S., Lee K. E., Haxby J. V., Martin A. (2002). Parallel visual motion processing streams for manipulable objects and human movements. *Neuron*.

[62] Rizzolatti G., Fogassi L., Gallese V. (2001). Neurophysiological mechanisms underlying the understanding and imitation of action. *Nature Reviews Neuroscience*.

[63] Allison T., Puce A., McCarthy G. (2000). Social perception from visual cues: Role of the STS region. *Trends in Cognitive Sciences*.

[64] Jellema T., Baker C. I., Wicker B., Perrett D. I. (2000). Neural representation for the perception of the intentionality of actions. *Brain and Cognition*.

[65] Perrett D. I., Harries M. H., Bevan R. (1989). Frameworks of Analysis for the Neural Representation of Animate Objects and Actions. *The Journal of Experimental Biology*.

[66] Arioli M., Perani D., Cappa S. (2018). Affective and cooperative social interactions modulate effective connectivity within and between the mirror and mentalizing systems. *Human Brain Mapping*.

[67] Canessa N., Alemanno F., Riva F. (2012). The neural bases of social intention understanding: The role of interaction goals. *PLoS ONE*.

[68] Winston J. S., Strange B. A., O'Doherty J., Dolan R. J. (2002). Automatic and intentional brain responses during evaluation of trustworthiness of faces. *Nature Neuroscience*.

[69] Bickart K. C., Dickerson B. C., Barrett L. F. (2014). The amygdala as a hub in brain networks that support social life. *Neuropsychologia*.

[70] Emery N. J., Capitanio J. P., Mason W. A., Machado C. J., Mendoza S. P., Amaral D. G. (2001). The effects of bilateral lesions of the amygdala on dyadic social interactions in rhesus monkeys (Macaca mulatta). *Behavioral Neuroscience*.

[71] Sporns O., Tononi G., Kötter R. (2005). The human connectome: a structural description of the human brain. *PLoS Computational Biology*.

[72] Wang Y., Metoki A., Alm K. H., Olson I. R. (2018). White matter pathways and social cognition. *Neuroscience & Biobehavioral Reviews*.

[73] Rokem A., Takemura H., Bock A. S. (2017). The visual white matter: The application of diffusion MRI and fiber tractography to vision science. *Journal of Vision*.

[74] Marstaller L., Burianová H., Reutens D. C. (2016). Individual differences in structural and functional connectivity predict speed of emotion discrimination. *Cortex*.

[75] Thomas C., Moya L., Avidan G. (2008). Reduction in white matter connectivity, revealed by diffusion tensor imaging, may account for age-related changes in face perception. *Cognitive Neuroscience*.

[76] Thomas C., Avidan G., Humphreys K., Jung K.-J., Gao F., Behrmann M. (2009). Reduced structural connectivity in ventral visual cortex in congenital prosopagnosia. *Nature Neuroscience*.

[77] Kamali A., Flanders A. E., Brody J., Hunter J. V., Hasan K. M. (2014). Tracing superior longitudinal fasciculus connectivity in the human brain using high resolution diffusion tensor tractography. *Brain Structure & Function*.

[78] Ethofer T., Bretscher J., Wiethoff S. (2013). Functional responses and structural connections of cortical areas for processing faces and voices in the superior temporal sulcus. *NeuroImage*.

[79] Gschwind M., Pourtois G., Schwartz S., Van De Ville D., Vuilleumier P. (2012). White-matter connectivity between face-responsive regions in the human brain. *Cerebral Cortex*.

[80] Heyes C. M., Frith C. D. (2014). The cultural evolution of mind reading. *Science*.

[81] Korman J., Voiklis J., Malle B. F. (2015). The social life of cognition. *Cognition*.

[82] Korkmaz B. (2011). Theory of Mind and Neurodevelopmental Disorders of Childhood. *Pediatric Research*.

[83] Canessa N., Gorini A., Cappa S. F. (2005). The effect of social content on deductive reasoning: An fMRI study. *Human Brain Mapping*.

[84] Canessa N., Pantaleo G., Crespi C., Gorini A., Cappa S. F. (2014). The impact of egocentric vs. allocentric agency attributions on the neural bases of reasoning about social rules. *Brain Research*.

[85] Mitchell R. L. C., Phillips L. H. (2015). The overlapping relationship between emotion perception and theory of mind. *Neuropsychologia*.

[86] Amodio D. M., Frith C. D. (2006). Meeting of minds: the medial frontal cortex and social cognition. *Nature Reviews Neuroscience*.

[87] Westby C. E. (2014). Social neuroscience and theory of mind. *Folia Phoniatrica et Logopaedica*.

[88] Molenberghs P., Johnson H., Henry J. D., Mattingley J. B. (2016). Understanding the minds of others: A neuroimaging meta-analysis. *Neuroscience & Biobehavioral Reviews*.

[89] Singer T., Lamm C. (2009). The social neuroscience of empathy. *Annals of the New York Academy of Sciences*.

[90] Bzdok D., Schilbach L., Vogeley K. (2012). Parsing the neural correlates of moral cognition: ALE meta-analysis on morality, theory of mind, and empathy. *Brain Structure & Function*.

[91] Shamay-Tsoory S. G., Harari H., Aharon-Peretz J., Levkovitz Y. (2010). The role of the orbitofrontal cortex in affective theory of mind deficits in criminal offenders with psychopathic tendencies. *Cortex*.

[92] Baron-Cohen S. (2001). Theory of Mind in Normal Development and Autism. *Prisme*.

[93] Wimmer H., Perner J. (1983). Beliefs about beliefs: representation and constraining function of wrong beliefs in young children's understanding of deception. *Cognition*.

[94] Baron‐Cohen S. (1989). The Autistic Child's Theory of Mind: a Case of Specific Developmental Delay. *Journal of Child Psychology and Psychiatry*.

[95] Mitchell J. P. (2006). Mentalizing and Marr: An information processing approach to the study of social cognition. *Brain Research*.

[96] Baron-Cohen S., Golan O., Ashwin E. (2009). Can emotion recognition be taught to children with autism spectrum conditions?. *Philosophical Transactions of the Royal Society B: Biological Sciences*.

[97] Decety J. (2010). The neurodevelopment of empathy in humans. *Developmental Neuroscience*.

[98] Gopnik A., Meltzoff A. N. (1997). Learning, Development, and Conceptual Change. *Words, Thoughts, and Theories*.

[99] Goldman A., Chater N., Hurley S. L. (2005). Imitation, Mind Reading, and Simulation. *Perspectives on Imitation: From Neuroscience to Social Science*.

[100] Proverbio A. M., Riva F., Paganelli L. (2011). Neural coding of cooperative vs. affective human interactions: 150 ms to code the action's purpose. *PLoS ONE*.

[101] Van Overwalle F., Baetens K. (2009). Understanding others' actions and goals by mirror and mentalizing systems: A meta-analysis. *NeuroImage*.

[102] Gallagher S., Hutto D. D., Racine T. P., Zlatev J., Sinha C., Itkonen E. (2008). Understanding Others through Primary Interaction and Narrative Practice. *Converging Evidence in Language and Communication Research (Celcr)*.

[103] Pfeiffer U. J., Timmermans B., Vogeley K., Frith C. D., Schilbach L. (2013). Towards a neuroscience of social interaction. *Frontiers in Human Neuroscience*.

[104] Parsons T. D. (2015). Virtual Reality for Enhanced Ecological Validity and Experimental Control in the Clinical, Affective and Social Neurosciences. *Frontiers in Human Neuroscience*.

[105] Renison B., Ponsford J., Testa R., Richardson B., Brownfield K. (2012). The ecological and construct validity of a newly developed measure of executive function: The virtual library task. *Journal of the International Neuropsychological Society*.

[106] Sebanz N., Bekkering H., Knoblich G. (2006). Joint action: Bodies and minds moving together. *Trends in Cognitive Sciences*.

[107] Schilbach L., Timmermans B., Reddy V. (2013). Toward a second-person neuroscience. *Behavioral and Brain Sciences*.

[108] Candidi M., Sacheli L. M., Era V., Canzano L., Tieri G., Aglioti S. M. (2017). Come together: Human-avatar on-line interactions boost joint-action performance in apraxic patients. *Social Cognitive and Affective Neuroscience*.

[109] Hamilton A. F. D. C., Grafton S. T. (2006). Goal representation in human anterior intraparietal sulcus. *The Journal of Neuroscience*.

[110] Buccino G., Vogt S., Ritzl A. (2004). Neural circuits underlying imitation learning of hand actions: an event-related fMRI study. *Neuron*.

[111] Bahnemann M., Dziobek I., Prehn K., Wolf I., Heekeren H. R. (2010). Sociotopy in the temporoparietal cortex: common versus distinct processes. *Social Cognitive and Affective Neuroscience*.

[112] Mar R. A. (2011). The neural bases of social cognition and story comprehension. *Annual Review of Psychology*.

[113] Spunt R. P., Adolphs R. (2014). Validating the Why/How contrast for functional MRI studies of Theory of Mind. *NeuroImage*.

[114] Spunt R. P., Lieberman M. D. (2012). An integrative model of the neural systems supporting the comprehension of observed emotional behavior. *NeuroImage*.

[115] Spunt R. P., Lieberman M. D. (2012). Dissociating modality-specific and supramodal neural systems for action understanding. *The Journal of Neuroscience*.

[116] Singer T., Seymour B., O'Doherty J., Kaube H., Dolan R. J., Frith C. D. (2004). Empathy for pain involves the affective but not sensory components of pain. *Science*.

[117] Singer T., Seymour B., O'Doherty J. P., Stephan K. E., Dolan R. J., Frith C. D. (2006). Empathic neural responses are modulated by the perceived fairness of others. *Nature*.

[118] Wicker B., Keysers C., Plailly J., Royet J.-P., Gallese V., Rizzolatti G. (2003). Both of us disgusted in My insula: The common neural basis of seeing and feeling disgust. *Neuron*.

[119] Keysers C., Wicker B., Gazzola V., Anton J.-L., Fogassi L., Gallese V. (2004). A touching sight: SII/PV activation during the observation and experience of touch. *Neuron*.

[120] Canessa N., Motterlini M., Di Dio C. (2009). Understanding others' regret: A fMRI study. *PLoS ONE*.

[121] Canessa N., Motterlini M., Alemanno F., Perani D., Cappa S. F. (2011). Learning from other people's experience: A neuroimaging study of decisional interactive-learning. *NeuroImage*.

[122] Lamm C., Decety J., Singer T. (2011). Meta-analytic evidence for common and distinct neural networks associated with directly experienced pain and empathy for pain. *NeuroImage*.

[123] Fan Y., Duncan N. W., de Greck M., Northoff G. (2011). Is there a core neural network in empathy? An fMRI based quantitative meta-analysis. *Neuroscience & Biobehavioral Reviews*.

[124] Carr L., Iacoboni M., Dubeaut M.-C., Mazziotta J. C., Lenzi G. L. (2003). Neural mechanisms of empathy in humans: a relay from neural systems for imitation to limbic areas. *Proceedings of the National Acadamy of Sciences of the United States of America*.

[125] Rymarczyk K., Zurawski L., Jankowiak-Siuda K., Szatkowska I. (2018). Neural correlates of facial mimicry: Simultaneous measurements of EMG and BOLD responses during perception of dynamic compared to static facial expressions. *Frontiers in Psychology*.

[126] Crespi C., Cerami C., Dodich A. (2014). Microstructural white matter correlates of emotion recognition impairment in Amyotrophic Lateral Sclerosis. *Cortex*.

[127] Crespi C., Cerami C., Dodich A. (2016). Microstructural correlates of emotional attribution impairment in non-demented patients with amyotrophic lateral sclerosis. *PLoS ONE*.

[128] Parkinson C., Wheatley T. (2014). Relating anatomical and social connectivity: White matter microstructure predicts emotional empathy. *Cerebral Cortex*.

[129] Hecht E. E., Gutman D. A., Preuss T. M., Sanchez M. M., Parr L. A., Rilling J. K. (2013). Process versus product in social learning: Comparative diffusion tensor imaging of neural systems for action execution-observation matching in macaques, chimpanzees, and humans. *Cerebral Cortex*.

[130] Catani M., Thiebaut de Schotten M. (2008). A diffusion tensor imaging tractography atlas for virtual in vivo dissections. *Cortex*.

[131] Von Der Heide R. J., Skipper L. M., Klobusicky E., Olson I. R. (2013). Dissecting the uncinate fasciculus: Disorders, controversies and a hypothesis. *Brain*.

[132] Catani M., Dell'Acqua F., Thiebaut de Schotten M. (2013). A revised limbic system model for memory, emotion and behaviour. *Neuroscience & Biobehavioral Reviews*.

[133] Downey L. E., Mahoney C. J., Buckley A. H. (2015). White matter tract signatures of impaired social cognition in frontotemporal lobar degeneration. *NeuroImage: Clinical*.

[134] Unger A., Alm K. H., Collins J. A., O'Leary J. M., Olson I. R. (2016). Variation in white matter connectivity predicts the ability to remember faces and discriminate their emotions. *Journal of the International Neuropsychological Society*.

[135] Genova H. M., Rajagopalan V., Chiaravalloti N., Binder A., Deluca J., Lengenfelder J. (2015). Facial affect recognition linked to damage in specific white matter tracts in traumatic brain injury. *Social Neuroscience*.

[136] Philippi C. L., Mehta S., Grabowski T., Adolphs R., Rudrauf D. (2009). Damage to association fiber tracts impairs recognition of the facial expression of emotion. *The Journal of Neuroscience*.

[137] Herbet G., Lafargue G., Bonnetblanc F., Moritz-Gasser S., Menjot De Champfleur N., Duffau H. (2014). Inferring a dual-stream model of mentalizing from associative white matter fibres disconnection. *Brain*.

[138] Levin H. S., Wilde E. A., Hanten G. (2011). Mental state attributions and diffusion tensor imaging after traumatic brain injury in children. *Developmental Neuropsychology*.

[139] Kana R. K., Libero L. E., Hu C. P., Deshpande H. D., Colburn J. S. (2014). Functional brain networks and white matter underlying theory-of-mind in autism. *Social Cognitive and Affective Neuroscience*.

[140] Herbet G., Lafargue G., Moritz-Gasser S., Bonnetblanc F., Duffau H. (2015). Interfering with the neural activity of mirror-related frontal areas impairs mentalistic inferences. *Brain Structure & Function*.

[141] Yordanova Y. N., Duffau H., Herbet G. (2017). Neural pathways subserving face-based mentalizing. *Brain Structure & Function*.

[142] Ruff C. C., Fehr E. (2014). The neurobiology of rewards and values in social decision making. *Nature Reviews Neuroscience*.

[143] Nash J. (1950). Equilibrium points in *N*-person games. *Proceedings of the National Acadamy of Sciences of the United States of America*.

[144] Camerer C. F. (2003). *Behavioral Game Theory: Experiments in Strategic Interaction*.

[145] Güth W., Schmittberger R., Schwarze B. (1982). An experimental analysis of ultimatum bargaining. *Journal of Economic Behavior & Organization*.

[146] Gale J., Binmore K. G., Samuelson L. (1995). Learning to be imperfect: the ultimatum game. *Games and Economic Behavior*.

[147] Nowak M. A., Page K. M., Sigmund K. (2000). Fairness versus reason in the ultimatum game. *Science*.

[148] Fehr E., Gintis H. (2007). Human motivation and social cooperation: Experimental and analytical foundations. *Annual Review of Sociology*.

[149] Henrich J., Boyd R., Bowles S. (2005). “Economic man” in cross-cultural perspective: Behavioral experiments in 15 small-scale societies. *Behavioral and Brain Sciences*.

[150] McCabe K. A., Rigdon M. L., Smith V. L. (2003). Positive reciprocity and intentions in trust games. *Journal of Economic Behavior & Organization*.

[151] Fehr E., Gächter S. (2002). Altruistic punishment in humans. *Nature*.

[152] Lerner J. S., Li Y., Valdesolo P., Kassam K. S. (2015). Emotion and decision making. *Annual Review of Psychology*.

[153] Keltner D., Lerner J. S., Fiske S. T., Gilbert D. T., Lindzey G. (2010). Emotion. *Handbook of Social Psychology*.

[154] Canessa N., Crespi C., Motterlini M. (2013). The functional and structural neural basis of individual differences in loss aversion. *The Journal of Neuroscience*.

[155] Canessa N., Crespi C., Baud-Bovy G. (2017). Neural markers of loss aversion in resting-state brain activity. *NeuroImage*.

[156] Bault N., Coricelli G., Rustichini A. (2008). Interdependent utilities: How social ranking affects choice behavior. *PLoS ONE*.

[157] Bault N., Joffily M., Rustichini A., Coricelli G. (2011). Medial prefrontal cortex and striatum mediate the influence of social comparison on the decision process. *Proceedings of the National Acadamy of Sciences of the United States of America*.

[158] Sanfey A. G., Rilling J. K., Aronson J. A., Nystrom L. E., Cohen J. D. (2003). The neural basis of economic decision-making in the Ultimatum Game. *Science*.

[159] Knoch D., Pascual-Leone A., Meyer K., Treyer V., Fehr E. (2006). Diminishing reciprocal fairness by disrupting the right prefrontal cortex. *Science*.

[160] Van't Wout M., Kahn R. S., Sanfey A. G., Aleman A. (2005). Repetitive transcranial magnetic stimulation over the right dorsolateral prefrontal cortex affects strategic decision-making. *NeuroReport*.

[161] De Quervain D. J.-F., Fischbacher U., Treyer V. (2004). The neural basis of altruistic punishment. *Science*.

[162] Rilling J. K., Gutman D. A., Zeh T. R., Pagnoni G., Berns G. S., Kilts C. D. (2002). A neural basis for social cooperation. *Neuron*.

[163] Fehr E., Camerer C. F. (2007). Social neuroeconomics: the neural circuitry of social preferences. *Trends in Cognitive Sciences*.

[164] Moll J., Krueger F., Zahn R., Pardini M., De Oliveira-Souza R., Grafman J. (2006). Human fronto-mesolimbic networks guide decisions about charitable donation. *Proceedings of the National Acadamy of Sciences of the United States of America*.

[165] Harbaugh W. T., Mayr U., Burghart D. R. (2007). Neural responses to taxation and voluntary giving reveal motives for charitable donations. *Science*.

[166] Izuma K., Saito D. N., Sadato N. (2010). Processing of the incentive for social approval in the ventral striatum during charitable donation. *Cognitive Neuroscience*.

[167] Mather M. (2016). The Affective Neuroscience of Aging. *Annual Review of Psychology*.

[168] Scheibe S., Carstensen L. L. (2010). Emotional Aging: Recent Findings and Future Trends. *The Journals of Gerontology Series B: Psychological Sciences and Social Sciences*.

[169] Kensinger E. A., Gutchess A, Duarte A. (2015). Memory for emotional and social information in adulthood and old age. *Handbook on the Cognitive Neuroscience of Human Memory*.

[170] Park D. C., Reuter-Lorenz P. (2009). The adaptive brain: aging and neurocognitive scaffolding. *Annual Review of Psychology*.

[171] Opitz P. C., Lee I. A., Gross J. J., Urry H. L. (2014). Fluid cognitive ability is a resource for successful emotion regulation in older and younger adults. *Frontiers in Psychology*.

[172] Krendl A. C., Heatherton T. F., Kensinger E. A. (2009). Aging Minds and Twisting Attitudes: An fMRI Investigation of Age Differences in Inhibiting Prejudice. *Psychology and Aging*.

[173] Kalokerinos E. K., von Hippel W., Henry J. D., Pachana N. (2015). Social Cognition and Aging. *Encyclopedia of Geropsychology*.

[174] Von Hippel W. (2007). Aging, executive functioning, and social control. *Current Directions in Psychological Science*.

[175] Salthouse T. (2012). Consequences of age-related cognitive declines. *Annual Review of Psychology*.

[176] Taaffe D. R., Harris T. B., Ferrucci L., Rowe J., Seeman T. E. (2000). Cross-sectional and prospective relationships of interleukin-6 and C-reactive protein with physical performance in elderly persons: MacArthur studies of successful aging. *The Journals of Gerontology. Series A, Biological Sciences and Medical Sciences*.

[177] Monge Z. A., Madden D. J. (2016). Linking cognitive and visual perceptual decline in healthy aging: The information degradation hypothesis. *Neuroscience & Biobehavioral Reviews*.

[178] Aizpurua A., Garcia-Bajos E., Migueles M. (2011). False recognition and source attribution for actions of an emotional event in older and younger adults. *Experimental Aging Research*.

[179] Mather M., Knight M. (2005). Goal-directed memory: the role of cognitive control in older adults' emotional memory. *Psychology and Aging*.

[180] Ruffman T., Henry J. D., Livingstone V., Phillips L. H. (2008). A meta-analytic review of emotion recognition and aging: implications for neuropsychological models of aging. *Neuroscience & Biobehavioral Reviews*.

[181] Calder A. J., Young A. W., Keane J., Dean M. (2000). Configural information in facial expression perception. *Journal of Experimental Psychology: Human Perception and Performance*.

[182] Circelli K. S., Clark U. S., Cronin-Golomb A. (2013). Visual scanning patterns and executive function in relation to facial emotion recognition in aging. *Aging, Neuropsychology, and Cognition*.

[183] Charles S. T., Carstensen L. L. (2010). Social and emotional aging. *Annual Review of Psychology*.

[184] Reed A. E., Chan L., Mikels J. A. (2014). Meta-analysis of the age-related positivity effect: Age differences in preferences for positive over negative information. *Psychology and Aging*.

[185] Isaacowitz D. M., Toner K., Neupert S. D. (2009). Use of Gaze for Real-Time Mood Regulation: Effects of Age and Attentional Functioning. *Psychology and Aging*.

[186] Reed A. E., Carstensen L. L. (2012). The theory behind the age-related positivity effect. *Frontiers in Psychology*.

[187] Grühn D., Rebucal K., Diehl M., Lumley M., Labouvie-Vief G. (2008). Empathy Across the Adult Lifespan: Longitudinal and Experience-Sampling Findings. *Emotion*.

[188] Reiter A. M. F., Kanske P., Eppinger B., Li S. C. (2017). The Aging of the Social Mind - Differential Effects on Components of Social Understanding. *Scientific Reports*.

[189] Moran J. M. (2013). Lifespan development: the effects of typical aging on theory of mind. *Behavioural Brain Research*.

[190] Carstensen L. L., Fung H. H., Charles S. T. (2003). Socioemotional selectivity theory and the regulation of emotion in the second half of life. *Motivation and Emotion*.

[191] Fjell A. M., Westlye L. T., Amlien I. (2009). High consistency of regional cortical thinning in aging across multiple samples. *Cerebral Cortex*.

[192] Erikson E. H. (1982). *The Life Cycle Completed, A Review*.

[193] Sze J. A., Gyurak A., Goodkind M. S., Levenson R. W. (2012). Greater emotional empathy and prosocial behavior in late life. *Emotion*.

[194] Weiner B., Graham S. (1989). Understanding the Motivational Role of Affect: Life-Span Research from an Attributional Perspective. *Cognition & Emotion*.

[195] Beadle J. N., Sheehan A. H., Dahlben B., Gutchess A. H. (2015). Aging, empathy, and prosociality. *The Journals of Gerontology B*.

[196] Davis M. H. (1983). Measuring individual differences in empathy: Evidence for a multidimensional approach. *Journal of Personality and Social Psychology*.

[197] Christidi F., Migliaccio R., Santamaría-García H., Santangelo G., Trojsi F. (2018). Social Cognition Dysfunctions in Neurodegenerative Diseases: Neuroanatomical Correlates and Clinical Implications. *Behavioural Neurology*.

[198] Fortier J., Besnard J., Allain P. (2018). Theory of mind, empathy and emotion perception in cortical and subcortical neurodegenerative diseases. *Revue Neurologique*.

[199] Realmuto S., Zummo L., Cerami C. (2015). Social cognition dysfunctions in patients with epilepsy: Evidence from patients with temporal lobe and idiopathic generalized epilepsies. *Epilepsy & Behavior*.

[200] Realmuto S., Dodich A., Meli R. (2018). Moral Cognition and Multiple Sclerosis: A Neuropsychological Study. *Archives of Clinical Neuropsychology*.

[201] Gorno-Tempini M. L., Hillis A. E., Weintraub S. (2011). Classification of primary progressive aphasia and its variants. *Neurology*.

[202] Rascovsky K., Hodges J. R., Knopman D., Mendez M. F., Kramer J. H., Neuhaus J. (2011). Sensitivity of revised diagnostic criteria for the behavioural variant of frontotemporal dementia. *Brain*.

[203] Cerami C., Dodich A., Canessa N. (2014). Emotional empathy in amyotrophic lateral sclerosis: a behavioural and voxel-based morphometry study. *Amyotrophic Lateral Sclerosis and Frontotemporal Degeneration*.

[204] Cerami C., Dodich A., Canessa N. (2014). Neural correlates of empathic impairment in the behavioral variant of frontotemporal dementia. *Alzheimer’s & Dementia*.

[205] Cerami C., Dodich A., Iannaccone S. (2015). Right limbic FDG-PET hypometabolism correlates with emotion recognition and attribution in probable behavioral variant of frontotemporal dementia patients. *PLoS ONE*.

[206] Caminiti S. P., Canessa N., Cerami C. (2015). Affective mentalizing and brain activity at rest in the behavioral variant of frontotemporal dementia. *NeuroImage: Clinical*.

[207] Gossink F., Schouws S., Krudop W. (2018). Social Cognition Differentiates Behavioral Variant Frontotemporal Dementia From Other Neurodegenerative Diseases and Psychiatric Disorders. *The American Journal of Geriatric Psychiatry*.

[208] Couto B., Manes F., Montañés P. (2013). Structural neuroimaging of social cognition in progressive non-fluent aphasia and behavioral variant of frontotemporal dementia. *Frontiers in Human Neuroscience*.

[209] Duval C., Bejanin A., Piolino P. (2012). Theory of mind impairments in patients with semantic dementia. *Brain*.

[210] Ibanez A., Manes F. (2012). Contextual social cognition and the behavioral variant of frontotemporal dementia. *Neurology*.

[211] Ferrari R., Kapogiannis D., Huey E. D., Momeni P. (2011). Ftd and als: A tale of two diseases. *Current Alzheimer Research*.

[212] Cerami C., Marcone A., Crespi C. (2015). Motor neuron dysfunctions in the frontotemporal lobar degeneration spectrum: A clinical and neurophysiological study. *Journal of the Neurological Sciences*.

[213] Crespi C., Dodich A., Cappa S. F. (2018). Multimodal MRI quantification of the common neurostructural bases within the FTD-ALS continuum. *Neurobiology of Aging*.

[214] Lattante S., Ciura S., Rouleau G. A., Kabashi E. (2015). Defining the genetic connection linking amyotrophic lateral sclerosis (ALS) with frontotemporal dementia (FTD). *Trends in Genetics*.

[215] Lillo P., Mioshi E., Burrell J. R., Kiernan M. C., Hodges J. R., Hornberger M. (2012). Grey and white matter changes across the amyotrophiclateral sclerosis-frontotemporal dementia continuum. *PLoS ONE*.

[216] Ling S. C., Polymenidou M., Cleveland D. W. (2013). Converging mechanisms in als and FTD: disrupted RNA and protein homeostasis. *Neuron*.

[217] Weishaupt J. H., Hyman T., Dikic I. (2016). Common Molecular Pathways in Amyotrophic Lateral Sclerosis and Frontotemporal Dementia. *Trends in Molecular Medicine*.

[218] Beeldman E., Raaphorst J., Twennaar M. K., De Visser M., Schmand B. A., De Haan R. J. (2016). The cognitive profile of ALS: A systematic review and meta-analysis update. *Journal of Neurology, Neurosurgery & Psychiatry*.

[219] Diehl-Schmid J., Pohl C., Ruprecht C., Wagenpfeil S., Foerstl H., Kurz A. (2007). The Ekman 60 Faces Test as a diagnostic instrument in frontotemporal dementia. *Archives of Clinical Neuropsychology*.

[220] Kessels R. P., Gerritsen L., Montagne B., Ackl N., Diehl J., Danek A. (2007). Recognition of Facial Expressions of Different Emotional Intensities in Patients with Frontotemporal Lobar Degeneration. *Behavioural Neurology*.

[221] Kumfor F., Piguet O. (2012). Disturbance of emotion processing in frontotemporal dementia: A synthesis of cognitive and neuroimaging findings. *Neuropsychology Review*.

[222] Torralva T., Roca M., Gleichgerrcht E., Bekinschtein T., Manes F. (2009). A neuropsychological battery to detect specific executive and social cognitive impairments in early frontotemporal dementia. *Brain*.

[223] Kumfor F., Hazelton J. L., De Winter F.-L., de Langavant L. C., Van den Stock J. (2017). Clinical studies of social neuroscience: A lesion model approach. *Neuroscience and Social Science: The Missing Link*.

[224] Zimmerman E. K., Eslinger P. J., Simmons Z., Barrett A. M. (2007). Emotional perception deficits in amyotrophic lateral sclerosis. *Cognitive and Behavioral Neurology*.

[225] Gray H. M., Tickle-Degnen L. (2010). A meta-analysis of performance on emotion recognition tasks in Parkinson's disease. *Neuropsychology*.

[226] Burrell J. R., Hodges J. R., Rowe J. B. (2014). Cognition in corticobasal syndrome and progressive supranuclear palsy: A review. *Movement Disorders*.

[227] Snowden J. S., Austin N. A., Sembi S., Thompson J. C., Craufurd D., Neary D. (2008). Emotion recognition in Huntington's disease and frontotemporal dementia. *Neuropsychologia*.

[228] Spoletini I., Marra C., Iulio F. D. (2008). Facial emotion recognition deficit in amnestic mild cognitive impairment and Alzheimer disease. *The American Journal of Geriatric Psychiatry*.

[229] Dodich A., Cerami C., Crespi C. (2016). Differential Impairment of Cognitive and Affective Mentalizing Abilities in Neurodegenerative Dementias: Evidence from Behavioral Variant of Frontotemporal Dementia, Alzheimer's Disease, and Mild Cognitive Impairment. *Journal of Alzheimer's Disease*.

[230] Dermody N., Wong S., Ahmed R., Piguet O., Hodges J. R., Irish M. (2016). Uncovering the Neural Bases of Cognitive and Affective Empathy Deficits in Alzheimer's Disease and the Behavioral-Variant of Frontotemporal Dementia. *Journal of Alzheimer's Disease*.

[231] Gleichgerrcht E., Ibáñez A., Roca M., Torralva T., Manes F. (2010). Decision-making cognition in neurodegenerative diseases. *Nature Reviews Neurology*.

[232] Moll J., Zahn R., de Oliveira-Souza R. (2011). Impairment of prosocial sentiments is associated with frontopolar and septal damage in frontotemporal dementia. *NeuroImage*.

[233] Melloni M., Billeke P., Baez S. (2016). Your perspective and my benefit: multiple lesion models of self-other integration strategies during social bargaining. *Brain*.

[234] Shalev I., Ebstein R. P. (2013). Frontiers in oxytocin science: From basic to practice. *Frontiers in Neuroscience*.

[235] Frommann N., Streit M., Wölwer W. (2003). Remediation of facial affect recognition impairments in patients with schizophrenia: A new training program. *Psychiatry Research*.

[236] Kayser N., Sarfati Y., Besche C., Hardy-Baylé M.-C. (2006). Elaboration of a rehabilitation method based on a pathogenetic hypothesis of “theory of mind” impairment in schizophrenia. *Neuropsychological Rehabilitation*.

[237] Vass E., Fekete Z., Simon V., Simon L. (2018). Interventions for the treatment of theory of mind deficits in schizophrenia: Systematic literature review. *Psychiatry Research*.

[238] Turner-Brown L. M., Perry T. D., Dichter G. S., Bodfish J. W., Penn D. L. (2008). Brief report: feasibility of social cognition and interaction training for adults with high functioning autism. *Journal of Autism and Developmental Disorders*.

[239] Berggren S., Fletcher-Watson S., Milenkovic N., Marschik P. B., Bölte S., Jonsson U. (2018). Emotion recognition training in autism spectrum disorder: A systematic review of challenges related to generalizability. *Developmental Neurorehabilitation*.

[240] Kurtz M. M., Richardson C. L. (2012). Social cognitive training for schizophrenia: A meta-analytic investigation of controlled research. *Schizophrenia Bulletin*.

[241] Fiszdon J. M., Reddy L. F. (2012). Review of social cognitive treatments for psychosis. *Clinical Psychology Review*.

[242] Dodich A., Cerami C., Canessa N. (2014). Emotion recognition from facial expressions: A normative study of the Ekman 60-Faces Test in the Italian population. *Neurological Sciences*.

[243] Dodich A., Cerami C., Canessa N. (2015). A novel task assessing intention and emotion attribution: Italian standardization and normative data of the Story-based Empathy Task. *Neurological Sciences*.

[244] Cassel A., McDonald S., Kelly M., Togher L. (2016). Learning from the minds of others: A review of social cognition treatments and their relevance to traumatic brain injury. *Neuropsychological Rehabilitation*.

